# Sputum Inflammatory Cell-Based Classification of Patients with Acute Exacerbation of Chronic Obstructive Pulmonary Disease

**DOI:** 10.1371/journal.pone.0057678

**Published:** 2013-05-31

**Authors:** Peng Gao, Jie Zhang, Xiaoyan He, Yuqiu Hao, Ke Wang, Peter G. Gibson

**Affiliations:** 1 Department of Respiratory Medicine, the Second Affiliated Hospital of Jilin University, Changchun, Jilin, China; 2 Department of Respiratory and Sleep Medicine, Hunter Medical Research Institute, John Hunter Hospital, Newcastle, New South Wales, Australia; 3 Jilin University-Woolcock Joint Research Centre for Airway Disease, Changchun, Jilin, China; 4 Changchun Central Hospital, Changchun, Jilin, China; French National Centre for Scientific Research, France

## Abstract

**Background:**

Patients with chronic obstructive pulmonary disease (COPD) commonly suffer from acute exacerbations (AECOPD) and display varying disease severity. However, there is no available biomarker for the classification of AECOPD. This study is aimed at investigating the sputum cellular profiles to classify patients with AECOPD.

**Methods:**

A total of 83 patients with AECOPD and 26 healthy controls were recruited. Their demographic and clinical characteristics were recorded, and their lung function was examined. The phenotypes of sputum inflammatory cells were characterised, and the concentrations of sputum and serum amyloid-A (SAA), C-reactive protein (CRP), interleukin-6 (IL-6), and matrix metalloproteinase-9 (MMP-9) were measured. Based on the sputum inflammatory cell profiles, individual patients were categorized into one of the four subgroups with inflammatory eosinophilic, neutrophilic, paucigranulocytic, and mixed granulocytic AECOPD. Most AECOPD patients were reevaluated within 12–14 months after discharge.

**Results:**

There were 10 (12%) eosinophilic, 36 (43%) neutrophilic, 5 (6%) mixed granulocytic, and 32 (39%) paucigranulocytic AECOPD patients. The patients with mixed granulocytic or neutrophilic AECOPD had a higher BODE score, more sputum inflammatory cells, lower lung function, and longer hospital stay, accompanied by higher concentrations of sputum MMP-9, IL-6 and CRP, and serum SAA, IL-6 and CRP. Notably, 83% of patients with neutrophilic AECOPD displayed evidence of bacterial infection and many of them responded poorly to standard therapies. In addition, patients with mixed granulocytic or neutrophilic stable COPD remained at lower lung functions and higher levels of inflammation.

**Conclusion:**

Patients with AECOPD display heterogeneous inflammation, and the profiles of sputum inflammatory cells may be used as valuable biomarkers for the classification of AECOPD patients.

## Introduction

Chronic obstructive pulmonary disease (COPD) is commonly accompanied by acute exacerbations (AECOPD), which contribute significantly to morbidity and mortality [1]. Currently, COPD is diagnosed based on the evidence of incompletely reversible airflow obstruction [1]. Furthermore, increased evidence has suggested that COPD is a multifactorial and multisystemic disease. Hence, multidimensional assessments are needed for the evaluation of disease severity. However, there are a few biomarkers available for the evaluation of AECOPD.

AECOPD is usually caused by pathogen infection-related inflammation and other insults. During the process of AECOPD, pro-inflammatory stimuli in the lung recruit inflammatory cells, such as neutrophils, eosinophils, macrophages, and lymphocytes, leading to the destruction of lung parenchyma and remodeling multiple components of the airway epithelial lumen. There are varying types of stimuli, which can recruit different types of inflammatory cells [2], [3]. In addition, the different inflammatory phenotypes are also clinically relevant due to potential differences in the response to therapeutic interventions. Indeed, previous studies have shown that the effects of treatments are different in COPD patients with different distributions of eosinophil infiltration or with acute exacerbation [2], [4], [5], and during exacerbations, and differing inflammatory patterns based on pathogens and biomarkers have been reported [2], [3]. Therefore, identification of the inflammatory phenotype in patients with AECOPD will be of great significance in understanding the disease process and in the management of patients with AECOPD.

Inflammatory cells can secrete pro-inflammatory cytokines, chemokines, and proteases contributing to the pathogenesis of AECOPD and the development of emphysema [6], [7]. Previous studies have shown that the concentrations of inflammatory mediators, such as serum amyloid A (SAA), C-reactive protein (CRP), Interleukin-6 (IL-6), and matrix metalloproteinase-9 (MMP-9), are correlated with the severity and are associated with poor prognosis of AECOPD [8]–[10]. However, the relationship among the levels of inflammatory mediators, the predominant type of inflammatory infiltrates in the lungs, and the degrees of functional impairment in the lung has not been clarified in patients with AECOPD. Moreover, how AECOPD patients with differently predominate inflammatory infiltrates respond to standard therapies is still not fully understood.

In this study, 83 AECOPD patients were recruited for examining the number of sputum inflammatory cells. Furthermore, these patients were stratified, according to the predominant type of inflammatory cell and their lung function and response to therapeutic treatments. Sputum and serum inflammatory mediators were examined to determine the potential association among the predominant type of inflammatory infiltrate, the levels of sputum and serum inflammatory mediators, and the degree of functional impairment in the lung. We tested the hypotheses that airway inflammation in AECOPD patients is heterogenous and can be classified by the predominant type of sputum inflammatory infiltrate, which are associated with the degrees of functional impairment in the lung.

## Materials and Methods

### Subjects

**Figure 1:**
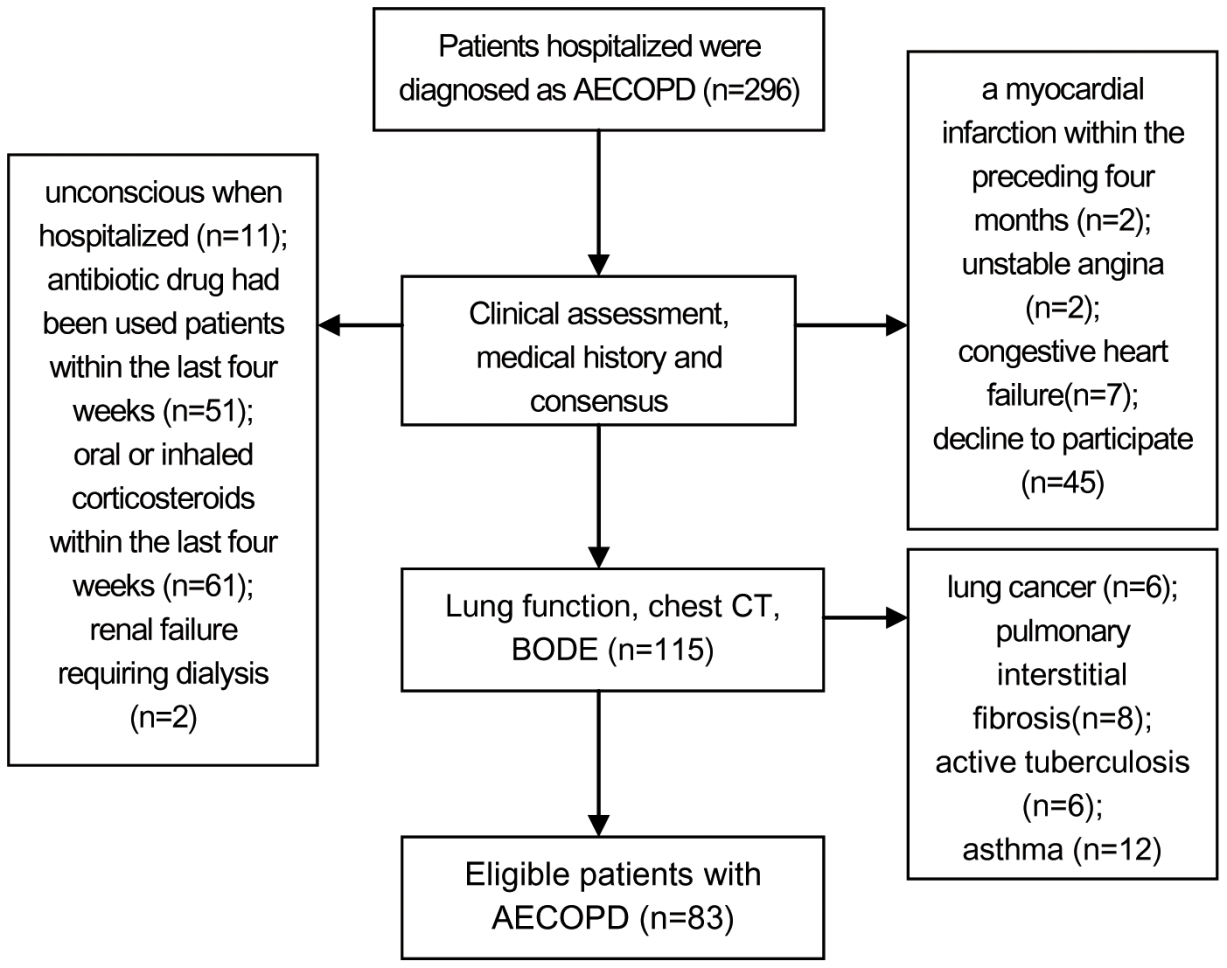
Strategies for screening patients with AECOPD.

A total of 296 patients with COPD were screened after they were admitted to the inpatient service of the Department of Respiratory Medicine of the Second Affiliated Hospital of Jilin University between March 2010 and June 2012, according to the strategies illustrated in Figure 1. Of these, 83 patients with AECOPD were recruited for this study. An additional 26 healthy control subjects who visited the outpatient service for regular health checks were recruited. All of the patients with AECOPD were diagnosed, according to the criteria established by the Global initiative for chronic Obstructive Lung Disease (GOLD) [1], and fulfilled the requirements of forced expiratory volume in one second (FEV_1_) <80% and FEV_1_/forced vital capacity (FVC) <70% following inhalation of a bronchodilator. Individual patients with a history of myocardial infarction, unstable angina, congestive heart failure, renal failure, cancer, pulmonary interstitial fibrosis, asthma, or currently active tuberculosis were excluded, and COPD patients had received antibiotics or corticosteroids during the past four weeks were also excluded. Furthermore, COPD patients who were unconscious or declined to participate were excluded from this study. According to the GOLD guidelines for the management of stable COPD [1], these patients were treated with the maintenance therapy, including 100–200 µg Salbutamol inhaler two to three times per day (n = 3), 4.5–12 µg Formoterol inhaler two times per day (n = 4), 50 µg Salmeterol inhaler one or two times per day (n = 11), 20–40 µg Ipratropine inhaler two or three times per day (n = 5), 18 µg Tiotropium inhaler one time per day (n = 16), orally with 200–400 mg Doxofylline (n = 18) two times per day, 200–300 mg Theophylline two times per day (n = 11), or 100–200 mg Aminophylline two or three times per day (n = 7). Written informed consent was obtained from individual subjects, and the experimental protocol was approved by the Medical Ethics Committee of the Second Affiliated Hospital of Jilin University, Changchun, Jilin, China.

### Study design

This was a cross-sectional and longitudinal study. After admission, individual patients were subjected to lung function examination [11] and sputum induction (SI) [12], routine sputum culture [13], and PCR analysis of sputum samples for the detection of viruses [14], including *rhinovirus, adenovirus, respiratory syncytial virus, and influenza virus A and B.* Their blood samples were obtained before treatment with antibiotics and corticosteroids. All of the patients were subjected to BODE evaluation [15], chest CT, and clinical assessments. Before discharge, the patients were examined by the six minute walk test (6MWT) [16].

Individual patients completed the clinical COPD questionnaire (CCQ) every day, and their clinical symptoms and signs were recorded. All the patients were treated intravenously with broad spectrum antibiotics (Amoxicillin/clavulanic acid, Ceftazidime, Cefoperazone Sodium/Sulbactam Sodium, Moxifloxacin) or orally with Cefuroxime, Moxifloxacin, and intravenously with 40 mg methylprednisolone daily for 7 days. The time to recovery for individual patients from an exacerbation was recorded, and recovery was defined as the CCQ score similar to that before exacerbation. The failure in treatment was defined as individuals who required continual antibiotic treatment after intensification of antibiotic therapy for 7 days. Some patients (61 cases) were re-examined within 12–18 months after discharge.

### Induction and processing of sputum samples

Induction and collection of sputum samples from individual patients were performed, as described previously [12]. Briefly, a high output ultrasonic nebulizer (ULTRA-NEB^TM^ Model 2000, De Vilbiss Healthcare, Somerset, PA, USA) with a Hans Rudolph 2-way valve box (Hans Rudolph, Shawnee, KS, USA) and tubing were used for delivering saline in doubling time periods from 0.5 sec to 4 min. Following baseline spirometry, individual patients were subjected to inhalation of 4×100 μg salbutamol via a pressurized metered dose inhaler and valved holding chamber, and were tested for post-bronchodilator spirometry 10 minutes later. These patients were nebulized for 30 seconds with a dose of 4.5% hypertonic saline or 0.9% saline, depending on their post-bronchodilator FEV_1_ ≥60% or <60% predicted. After the nebulization, the patients were encouraged to cough and expectorate for the collection of sputum samples. Then, they were tested for the FEV_1_. If their FEV_1_ percentage fall was less than 15%, they were nebulized again. The induction continued in increments up to a cumulative time of 15.5 minutes (30 sec, 1 min, 2 min, and 3×4 min intervals). If the FEV_1_ fell by more than 15% at any time during the induction, the patient was provided with 2×100 μg salbutamol via a spacer and re-tested for the FEV_1_ 10 minutes later. The criteria for stopping the sputum induction included a drop of 15% FEV_1_ more than two occasions, patient's request or symptoms, and investigator's discretion.

The collected sputum samples were placed onto a clean open Petri dish and the mucus clumps in the samples were separated from saliva using a forceps. The separated mucus clumps (0.1–1 ml) were mixed with four volumes of diluted dithiothreitol (Sputolysin) in a 15 ml tube and incubated at 37°C in a water bath for 30 minutes with gently shaking. Subsequently, the samples were mixed with equal volume of PBS and filtered through a nylon filter (60 μm) apparatus. The numbers of cells were counted and after centrifugation, the supernatants were stored at −80°C. The cell pellet was resuspended in PBS and adjusted to a final concentration of 1×10^6^/ml. The cell suspension was subjected to cytospins, and the cells were stained with May-Grunwald Giemsa and Chromotrope 2R, followed by examination under a light microscope. A sputum sample was considered to be inadequate when the percentage of squamous cells was >80%.

### Stratification of AECOPD patients

All of the AECOPD patients were stratified, according to the number of neutrophils (>61%) and eosinophils (>2.5%) in the sputum samples, which were the cutoff values of the 95th percentile of healthy controls, respectively [17]. Individual patients were classified into the eosinophilic COPD (EO) with sputum eosinophils >2.5% of total cells, the neutrophilic COPD (NE) with neutrophils >61%, the paucigranulocytic COPD (PA) with eosinophils ≤2.5% and neutrophils ≤61%, and the mixed granulocytic COPD (MC) with eosinophils >2.5% and neutrophils >61%.

### Measurement of inflammatory mediators

The concentrations of serum and sputum SAA (56-SAA HU-E02), CRP (DCRP00), MMP-9 (DMP900), and IL-6 (D6050; R&D Systems; USA) were determined by ELISA using specific kits, according to the manufacturers' instructions (ALPCO; Netherlands, R&D Systems; USA) [18]–[20]. The detection limitations were 200 ng/ml for SAA, 50 ng/ml for CRP, 20 ng/ml for MMP-9, and 300 pg/ml for IL-6.

### Statistics

The distribution of individual groups of data was analyzed. SPSS17.0 was used for statistical analysis. If the data were normally distributed, they are expressed as the mean and standard deviation (SD) and analyzed by ANOVA test with Bonferroni correction or Student's *t*-test. If the data were not normally distributed, they were reported as the median and interquartile range (IQR) and analyzed by Kruskall-Wallis test or the Mann-Whitney *U* test. Categorical variables were analyzed by Chi squared test. All of the statistical analyses were performed using SPSS17.0 software. A p value of <0.05 was considered statistically significant.

## Results

### Studying patients

To determine the inflammatory cellular phenotypes, a total of 296 patients with COPD were screened and 83 patients with AECOPD were included. There was no significant difference in age, smoking status, and the amounts of cigarettes between the AECOPD patients and healthy controls (Table 1). In comparison with that in the healthy controls, AECOPD patients were comprised of significantly more female subjects and had lower values of BMI. Furthermore, AECOPD patients displayed significantly lower lung function. In addition, sputum samples were successfully induced in 88% of AECOPD patients and 81% of healthy controls and AECOPD patients produced an average of 19 ml of sputum in 40 min following stimulation. Characterisation of sputum inflammatory infiltrates indicated a significantly greater number of total inflammatory infiltrates, neutrophils, eosinophils, macrophages, and lymphocytes in the sputum samples from AECOPD patients than from the healthy controls and that there were huge variations in the number of each type of inflammatory infiltrates in those patients. Spirometry revealed that patients had moderate to very-severe COPD (GOLD II–IV). However, there were 10% (8/83) of AECOPD patients who were not sustainable for the 6 MWT, due to dyspnea (6 cases) or leg pain (2 cases).

**Table 1:** The demographic and clinical characteristics of subjects.

	AECOPD	control
Subjects n	83	26
Age (years)	63.23±11.42	60.44±13.42
Male/female	61/22*	25/1
BMI	21.6±4.8*	24.6±3.7
Current smoker yes/no	40/43	9/17
Pack-yrs	19.11±11.92	15.32±13.85
Post-bronchodilator FEV_1_/FVC (%)	0.58±0.08*	0.83±0.05
Post-bronchodilator FEV_1_(L)	1.22±0.51*	3.15±0.88
Post-bronchodilator FEV_1_%pred (%)	39.8±14.7*	93.0±14.7
Total cell count (10^6^/mL)	6.1(2.0–23.8)*	1.3(1.2–1.8)
Neutrophils (10^6^/mL)	2.2(0.4–20)*	0.5(0.4–0.8)
Eosinophils (10^6^/mL)	0.03(0–0.3)*	0.0(0.0–0.01)
Macrophages (10^6^/mL)	1.4(0.7–3.6)*	0.8(0.6–1.0)
Lymphocytes (10^6^/mL)	0.1(0.0–0.4)*	0.02(0.01–0.04)
Epithelials (10^6^/mL)	0.6(0.3–1.0)	0.8(0.2–1.8)
Squamous cells (10^6^/mL)	0.4(0.2–1.0)	0.7(0.3–1.0)
GOLD I	0	n/a
GOLD II	12	n/a
GOLD III	51	n/a
GOLD IV	20	n/a

Data are expressed as the mean ± SD or median (IQR). The difference between groups was analyzed by Student *t*-test, the Mann-Whitney *U* test or Chi square. *P<0.05 vs. the control.

### Inflammatory phenotypes of AECOPD patients

We further stratified patients, according to the number of each type of inflammatory infiltrates in the sputum samples, and divided the patients into the NE (36 cases, 43%), PA (32, 39%), EO (10, 12%), and MC (5, 6%) groups, respectively (Table 2). There was significant difference in the number of sputum inflammatory infiltrates among these groups of patients. There were a significantly increased number of total inflammatory infiltrates, neutrophils, and eosinophils in the sputum samples from the NE, EO and MC groups of patients compared with that in the PA group of patients. Similarly, the number of eosinophils in the sputum samples from the EO and MC groups was significantly greater than that in the NE and PA groups of patients. In addition, the MC group of patients had significantly more numbers of macrophages in the sputum samples, but there was no significant difference in the number of lymphocytes, epithelial, and squamous cells in the sputum samples among these groups of patients. Interestingly, the MC and NE groups of patients had significantly greater numbers of blood leukocytes and neutrophils than that in the EO and PA groups of patients, and the MC and EO groups of patients had greater numbers of blood eosinophils than that in the NE and PA groups of patients. These data indicated that different groups of patients had varying numbers and types of inflammatory cells in the sputum samples.

**Table 2:** Clinical characteristics of AECOPD patients.

	Eosinophilic	Neutrophilic	Mixed granulocytic	Paucigranulocytic
N	10	36	5	32
Age (years)	64.8±11.9	65.9±10.5	66.4±11.4	62.8±10.0
BODE score	6(5–6.25)^+^	5.5(4–7)^+^	6(4.5–7.5)^+^	1(0.3–2.8)
GOLD II	0	5	0	7
GOLD III	6	19	1	25
GOLD IV	4	12	4	0
Post-FEV_1_ (L)	0.99±0.20^‡^	1.24±0.52	0.61±0.06^‡‖^	1.34±0.53
Post-FEV_1_/pred (%)	31.4±5.1^‡^	38.5±9.8^‡^	22.8±5.0^‡^	46.7±18.3
FEV_1_/FVC (%)	57.3±12.3	59.3±7.0	56.1±5.9	58.7±8.5
Bacteria	1 (10%)	31 (86%)^‡§¶^	2(40%)	2(6%)
Virus	3 (30%)	7 (19%)	1(20%)	5(16%)
Volume of sputum (mL)	12(6.5–16)	23(16–41)*^+^	22(17–39)*^+^	15(7.5–21)
symptom recovery time (days)	6.0 (4.8–9.3)	11.0 (9–15)*^+^	12.0 (6.5–20)*^+^	5.5 (1.3–8)
Lengths of hospital stay (days)	8.0 (7.8–11.5)	12 (10.3–16.8)*^+^	16 (7.5–22.5)*^+^	8.5 (5.3–11)
intensification of drug therapy	1 (10%)	13 (36%)^‡^	2 (40%)	2 (6.3%)
Blood leukocytes (10^9^/L)	10.5(8.5–14.2)	15.3(9.8–21)*^+^	16.1(10.1–18.4)*^+^	11.4(8.9–14.1)
Blood neutrophils (10^9^/L)	7.1(5.5–9.8)	12.7(8.2–16.2)*^+^	12.0(8.5–15.9)*^+^	7.4(6.3–11.5)
Blood eosinophils (10^9^/L)	0.96(0.74–1.62)^+†^	0.24(0.1–0.41)	0.98(0.86–1.45)^+†^	0.13(0–0.35)
Total cell count (10^6^/mL)	3.6(2.1–5.6)^+^	23.3(10.7–32.8)^+^*	25.5(19.5–44.9)^+^*	1.3(0.8–3.9)
neutrophils (10^6^/mL)	1.0(0.7–1.5)^+^	20.8(9.7–27.2)^+^*	19.4(15.5–18.9)^+^*	0.3(0.1–0.7)
eosinophils (10^6^/mL)	0.4(0.2–1.0)^+†^	0.1(0.0–0.3)^+^	2.2(1.2–2.9)^+†^	0.0(0.0–0.0)
macrophages (10^6^/mL)	1.2(0.6–2.8)	1.9(0.9–4.5)	4.5(2.0–13.5)^+^*^†^	1.2(0.6–2.9)
lymphocytes (10^6^/mL)	0.0(0.0–0.02)	0.0(0.0–0.66)	0.0(0.0–0.02)	0.0(0.0–0.01)
epithelial cells (10^6^/mL)	0.3(0.1–0.8)	0.7(0.4–1.2)	1.2(0.45–1.8)	0.4(0.2–0.8)
Squamous cells (10^6^/mL)	0.1(0.0–0.5)	0.6(0.2–1.0)	0.2(0.15–1.6)	0.4(0.2–1.0)

Data are expressed as mean ± SD or median (IQR). The difference among groups was determined by ANOVA, Kruskall-Wallis, Mann-Whitney *U* test or Chi square. *P<0.01 vs. the Eosinophilic AECOPD; ^+^P<0.01 vs. the Paucigranulocytic AECOPD; ^†^P<0.01 vs. the Neutrophilic AECOPD; ^‡^P<0.05 vs. the Paucigranulocytic AECOPD; ^‖^P<0.05 vs. the Neutrophilic AECOPD; ^§^P<0.05 vs. the Eosinophilic AECOPD; ^¶^P<0.05 vs. the Mixed granulocytic AECOPD.

### Clinical characteristics in different groups of AECOPD patients

The results of clinical assessments in these four groups of AECOPD patients are summarised in Table 2. There were no significant difference in age and FEV_1_/FVC (%) among these groups of patients (Age: F = 0.54, P = 0.657; FEV_1_/FVC (%): F = 0.303, P = 0.823). The MC, NE, and EO groups of patients had significantly lower predicted FEV_1_% than PA (P<0.05), and the MC group of patients had significantly lower values of the post-FEV_1_ than those in the NE and PA groups (P<0.05). There was significant difference in the values of BODE scores and GOLD stages among these groups of patients. The EO, NE, and MC groups of patients had significantly higher BODE scores than that of the PA group of patients. The MC group of patients had the highest GOLD stages, followed by the EO, NE, and PA groups of patients. The NE and MC groups of patients produced more amounts of sputum than those in the EO and PA groups. Consistently, microbiological analysis indicated that 19% and 43% of AECOPD patients had evidence of virus and bacterial infection, respectively, and patients in the NE and MC groups had significantly more frequent evidence of bacterial infection than those in the EO and PA groups. These data suggest that different groups of patients had varying types of inflammatory infiltrates and disease severities.

### Inflammatory mediators in different groups of AECOPD patients

Next, we measured the concentrations of sputum and serum inflammatory mediators in the different groups of AECOPD patients. We found that the concentrations of sputum and serum CRP, IL-6, MMP-9, and serum SAA in AECOPD patients were significantly higher than that in the controls (P<0.01, Table 3). The highest concentrations of sputum CRP, sputum MMP-9, and serum IL-6 were detected in the MC group of patients, followed by the NE group (Table 3). The levels of sputum and serum CRP in the MC and NE groups of patients were significantly higher than that in the EO and PA groups of patients (P<0.05). The concentrations of sputum, but not serum, MMP-9 in the MC and NE groups were significantly higher than that in the PA (P<0.05). Similarly, the levels of sputum IL-6 in the MC and NE groups were significantly higher than that in the PA and EO groups, and serum IL-6 were higher than that in the PA group (P<0.05). The concentrations of serum SAA in the MC and NE groups were higher than that in the EO and PA groups (P<0.05), while the levels of SAA in sputum supernatant remained below the detection threshold of the assays. Thus, patients in the MC and NE groups had more severe inflammation.

**Table 3:** The levels of serum and sputum inflammatory mediators in AECOPD patients.

	Eosinophilic	Neutrophilic	Mixed granulocytic	Paucigranulocytic	control
Blood CRP (mg/L)	10(8.4–13.2)	16(12–19)^+^*	14.8(14.3–18.2)^+^*	12(7.3–15)	0.83(0.5–1.6)
Sputum CRP (ug/L)	48(24–112)	145(78–170)^+^*	199(175–237)^+^*^¶^	22(11–40)	7(3.8–16)
Blood MMP- 9 (ng/mL)	1030(406–1497)	750(516–1161)	1760(828–4810)	680(385–1427)	355(165–648)
Sputum MM P-9 (ng/mL)	528(338–3159)	1836(1045–3891)^+^	4914(3140–6390)^+^*^¶^	930(293–2117)	392(93–804)
Blood IL-6 (pg/mL)	19(12–32)	31(17–87)^+^	125(47–132)^+^*^¶^	16(7.0–32)	5.7(3.4–7.7)
Sputum IL-6 (pg/mL)	362(268–770)	918(447–1372)^+^*	2541(765–4890)^+^*	459(167–1089)	48(31–140)
Blood SAA (mg/L)	36(27–44)	84(64–116)^+^*	142(52–153)^+^*	32(23–42)	3.8(2.9–7.5)

Data are expressed as median (IQR). The difference among groups was determined by Kruskall-Wallis and Mann-Whitney *U* test. *P<0.05 vs. the Eosinophilic; ^+^P<0.05 vs. the Paucigranulocytic; ^¶^P<0.05 vs. the Neutrophilic; All of the patient groups were significantly higher than that in the controls (P<0.01).

### Therapeutic responses of different groups of AECOPD patients

Following treatment with antibiotics and methylprednisolone as well as other supportive medicines, we found that 65 out of 83 AECOPD patients recovered and that 18 patients failed the treatment and required continual antibiotic therapy. While most patients responded to standard therapies, there were significantly more patients in the NE group who required intensification of drug therapy (Table 2). Similarly, patients in the NE and MC groups spent significantly longer time for recovery and stay in the hospital than those in the EO and PA groups (Table 2). Apparently, the MC and NE groups of patients had more severe AECOPD and poorer responses to the standard therapies.

We followed up 61 out of 83 AECOPD patients for about 14 months (Table 4). Those patients with stable COPD were continually treated with maintenance therapies and remained in the same group, except for two patients from the EO to PA, two patients from the PA to NE group, and 1 patient from the NE to PA group. The kappa statistic (95% confidence interval) was 0.87 (0.76–0.98) (P<0.01), indicating substantial agreement in classifications between the visits. Analysis of the different groups of patients revealed that the BODE scores and GOLD stages in all groups of patients were reduced, but the BODE scores and GOLD stages in the NE and MC groups were significantly greater than that in the EO and PA groups of patients. Furthermore, all groups of patients had slightly improved lung function, and the percentages of post-FEV_1_ to predicted value in the NE and MC groups of patients were significantly less than that in the EO and PA groups of patients. Similarly, the amounts of sputum from the NE and MC groups were significantly greater than that in the EO and PA groups of patients. Laboratory tests indicated that all groups of patients had blood leukocytes, eosinophils and macrophages in normal ranges, although the number of leukocytes in the NE and MC was greater than that in the EO and PA groups. In contrast, the number of eosinophils in the EO and MC groups was greater than that in the NE and PA groups of patients and there was no significant difference in the number of blood neutrophils among the different groups of patients. Similarly, the NE and MC groups of patients had significantly greater numbers of inflammatory infiltrates and neutrophils in the sputum samples, while the EO and MC groups of patients had significantly greater numbers of eosinophils in the sputum samples. Finally, significantly higher concentrations of sputum CRP, MMP-9, and IL-6 and serum CRP, IL-6, and SAA were detected in the NE and MC groups of patients, as compared with that in the EO and PA groups of patients (Table 5). Apparently, the NE and MC groups of patients with stable COPD still had higher degrees of inflammation.

**Table 4:** Clinical characteristics of patients with stable COPD.

	Eosinophilic	Neutrophilic	Mixed granulocytic	Paucigranulocytic
N	5	29	3	24
Age (years)	66.0±13.0	65.4±11.2	60.3±10.8	62.8±10.1
BODE score	1.0(0.0–1.0)	3.0(2.0–4.8)*^¶^	3.0(3.0–5.0)*^¶^	0.0(0.0–1.0)
GOLD I	2	2	0	2
GOLD II	2	5	0	11
GOLD III	1	15	1	11
GOLD IV	0	7	2	0
Post-FEV_1_ (L)	1.33±0.42	1.28±0.44^‡^	0.78±0.05^§‡^	1.39±0.49
Post-FEV_1_/pred (%)	43.3±16.0	40.8±7.6^‡^	30.0±4.1^‡^	49.0±17.4
FEV_1_/FVC (%)	61.1±9.3	60.4±8.9	58.4±7.3	62.4±7.6
Volume of sputum (mL)	4(2–7)	13(9–17)*^¶^	14(9–18)*^¶^	6(2.5–10)
Blood leukocytes (10^9^/L)	6.4(5.3–7.8)	8.3(6.7–9.2)*^¶^	7.8(7.0–9.2)^‡*^	7.2(6.2–9.4)
Blood neutrophils (10^9^/L)	4.3(3.4–5.1)	5.1(3.5–6.2)	4.8(4.1–5.3)	4.9(3.9–5.7)
Blood eosinophils (10^9^/L)	0.67(0.54–0.8)^†¶^	0.17(0.0–0.35)	0.7(0.53–0.9)^†¶^	0.11(0.0–0.28)
Total cell count (10^6^/mL)	1.4(0.8–3.2)	15.3(7.2–21.1)*^¶^	16.4(10.6–19.7)*^¶^	1.0(0.5–4.2)
Neutrophils (10^6^/mL)	0.7(0.4–1.1)	10.3(6.5–14.2)*^¶^	12.1(7.4–16.3)*^¶^	0.2(0.1–0.6)
eosinophils (10^6^/mL)	0.3(0.2–0.9)^¶†^	0.1(0.0–0.2)	1.8(0.9–1.9)^¶†^	0.0(0.0–0.1)
macrophages (10^6^/mL)	0.9(0.3–2.1)	1.4(0.3–2.9)	2.2(0.2–11.4)	0.7(0.2–1.3)
lymphocytes (10^6^/mL)	0.0(0.0–0.03)	0.0(0.0–0.42)	0.0(0.0–0.12)	0.0(0.0–0.02)
epithelial cells (10^6^/mL)	0.8(0.4–1.2)	0.9(0.3–1.7)	0.9(0.5–1.4)	1.6(0.7–1.7)
Squamous cells (10^6^/mL)	0.3(0.0–0.7)	0.8(0.2–1.4)	0.7(0.3–1.9)	1.2(0.6–1.1)

Data are expressed as mean ± SD or median (IQR). The difference among groups was determined by ANOVA, Kruskall Wallis, Mann-Whitney *U* test or Chi square. *P<0.01 vs. the Eosinophilic COPD; ^¶^P<0.01 vs. the Paucigranulocytic COPD; ^†^P<0.01 vs. the Neutrophilic COPD; ^‡^P<0.05 vs. the Paucigranulocytic COPD; ^§^P<0.05 vs. the Neutrophilic COPD.

**Table 5:** The levels of serum and sputum inflammatory mediators in stable COPD patients.

	Eosinophilic	Neutrophilic	Mixed granulocytic	Paucigranulocytic	control
Blood CRP (mg/L)	3.8(3–4.7)^∧^	5.6(3.9–7.5)^§**¶**∧^	7.8(7.5–9.2)^§¶∧^	3.9(3–5.2)^∧^	0.83(0.5–1.6)
Sputum CRP (ug/L)	7.3(5.5–14)	48(22–78)^¶^*^∧^	98(54–129)^¶^*^∧^	9.4(6.4–22)	7(3.8–16)
Blood MMP-9 (ng/mL)	375(179–729)	346(220–471)	426(325–672)	420(139–951)	355(165–648)
Sputum MMP-9 (ng/mL)	444(287–1171)^∧^	694(399–1580)^¶^*^∧^	1847(1526–2648)^†¶^*^∧^	430(232–910)^∧^	392(93–804)
Blood IL-6 (pg/mL)	5(1.8–7.2)	10(7.1–24)^¶^*^∧^	21(1.6–30)^¶^*^∧^	6.2(2.7–8.8)	5.7(3.4–7.7)
Sputum IL-6 (pg/mL)	147(107–487)^∧^	342(149–620)^¶^*^∧^	1328(373–3527)^‡¶^*^∧^	111(52–381)^∧^	48(31–140)
Blood SAA (mg/L)	8.4(5.1–13)^∧^	22(14–41)^¶^*^∧^	22(17–43)^¶^*^∧^	11(8–13.7)^∧^	3.8(2.9–7.5)

Data are expressed as median (IQR). The difference among groups was determined by Kruskall-Wallis and Mann-Whitney *U* test. *P<0.01 vs. the Eosinophilic COPD; ^¶^P<0.01 vs. the Paucigranulocytic COPD; ^†^P<0.01 vs. the Neutrophilic COPD; ^§^P<0.05 vs. the Eosinophilic COPD; ^‡^P<0.05 vs. the Neutrophilic COPD; ^∧^P<0.05 vs. the control.

## Discussion

In this study, we found that 61% of AECOPD patients not only had impairment of lung function, but also had varying levels of airway inflammation, accompanied by various types of inflammatory infiltrates in their lungs. These data are consistent with previous observations [2], [3] and support the notion that any reason-mediated local inflammation can trigger the development of AECOPD in COPD patients [1]. Interestingly, 39% of AECOPD patients (termed a paucigranulocytic pattern) had their airway inflammatory infiltrates similar to that in the healthy controls, but they had significantly higher levels of sputum IL-6 and MMP-9 and serum CPR and SAA. These observations suggest that inflammation in the non-lung organs may cause airway responses that promote higher levels of inflammatory mediators in the lungs, leading to the development of AECOPD. Indeed, these patients had shorter recovery time and hospital stay, implicating that these patients responded well to the standard therapies. Although the precise factors that trigger the development of AECOPD remain to be investigated, these patients may only require standard therapies.

Characterisation of sputum inflammatory cells in majority of AECOPD patients revealed that sputum inflammatory cells were comprised predominantly of neutrophils, eosinophils, and macrophages in the lungs. Further stratification of patients, according to the predominant types of inflammatory infiltrates in their sputum, indicated that these patients were classified into eosinophilic, neutrophilic, and mixed granulocytic groups. The NE group had more patients with bacterial infection and produced more sputum, accompanied by higher levels of sputum and serum inflammatory mediators. As a result, some patients took significantly longer time for recovery and hospital stay and more patients required intensification of drug therapy, particularly for those who had been infected with drug-resistant bacteria. Apparently, the NE group of patients usually displayed severe AECOPD and responded poorly to standard therapies. Because control of bacterial infection in the lung is crucial for the recovery of lung function [21], [22], it is important to determine the infected bacteria and their susceptibility to antibiotics to eliminate the infection effectively. Given that many patients in the NE group had higher levels of inflammatory mediators, regular treatment with antibiotics may be valuable for preventing the development of AECOPD patients.

The MC group of patients displayed elevated numbers of sputum neutrophils and eosinophils, more severe impairment of lung function and disease severity, accompanied by higher levels of sputum and serum inflammatory mediators. Like patients in the NE group, some patients in the MC group also had evidence of bacterial infection and responded poorly to the standard therapies, accompanied by higher levels of sputum and serum inflammatory mediators at their stable stage. As a result, they had the longest time for recovery and hospital stay. In contrast, the EO group of patients with predominant eosinophil infiltrates in the lungs had lower levels of sputum and serum inflammatory mediators and responded well to the standard therapies, accompanied by shorter time of recovery and hospital stay. However, patients in the EO group, like those in the MC group, usually had severe impairment of lung function. Apparently, elevated eosinophil infiltration in the lungs is associated with severe impairment of lung function. Indeed, eosinophilic inflammation is present in about 20%–40% of patients with COPD [2], [4], [5]. Increased number of eosinophils were detected even in patients with stable COPD [23]. Hence, characterisation of eosinophils in the lungs of AECOPD patients may be valuable for the design of therapies for AECOPD [2]. We are interested in further investigation of how eosinophil infiltration contributes to the impairment of lung function.

Currently, functional criteria, clinical symptoms, and measurements have been used for the classification of AECOPD patients [1]. Although sputum neutrophil counts and the levels of serum CRP are good biomarkers for evaluating the severity of AECOPD [8], [9], other biomarkers, such as serum cytokines and SAA, are also important for the identification and management of AECOPD [10]. We employed a range of mediators and sputum inflammatory cells to classify AECOPD patients into four groups and found that patients in individual groups had unique clinical characteristics, similar to that of a previous report [3]. We found that the levels of serum CRP, IL-6, and SAA and sputum MMP-9, CRP, and IL-6, together with the predominant type of inflammatory cells, were excellent biomarkers for judging the severity of AECOPD in this population. Our initial observations suggest that in an inflammatory exacerbation of COPD, like in an acute exacerbation of asthma, both the intensity and the pattern of the inflammatory response are key determinants of the severity of the exacerbation [17]. The various types of inflammatory infiltrates in the lungs of AECOPD patients also suggest that diversely causative factors can trigger the development of AECOPD. Therefore, our findings may provide a new basis for the clinical management of AECOPD and study of the pathogenic mechanisms of AECOPD.

## Conclusion

In this study, we employed sputum inflammatory cells and other inflammatory mediators to classify AECOPD patients into four groups. We found that AECOPD patients in individual groups had unique clinical characteristics and inflammatory mediator profile as well as microbial infection. Furthermore, AECOPD patients in the different groups displayed various responses to the standard therapies and different inflammatory status at a stable stage. Therefore, this inflammatory phenotype classification is not only useful for the management of AECOPD patients, but also valuable for investigating the pathogenesis of AECOPD. We recognised that our study had limitations of small sample size at only a few time points and lack of functional examination of inflammatory cells. Thus, further continual studies on the pathogenesis of AECOPD and the function of inflammatory cells of a bigger population are warranted.
